# Developmental Commonalities between Object and Face Recognition in Adolescence

**DOI:** 10.3389/fpsyg.2016.00385

**Published:** 2016-03-15

**Authors:** Martin Jüttner, Elley Wakui, Dean Petters, Jules Davidoff

**Affiliations:** ^1^Department of Psychology, School of Life and Health Sciences, Aston UniversityBirmingham, UK; ^2^School of Psychology, University of East LondonLondon, UK; ^3^Department of Psychology, Birmingham City UniversityBirmingham, UK; ^4^Department of Psychology, Goldsmiths, University of LondonLondon, UK

**Keywords:** development, object recognition, face recognition, categorical, metric, part, configural, holistic

## Abstract

In the visual perception literature, the recognition of faces has often been contrasted with that of non-face objects, in terms of differences with regard to the role of parts, part relations and holistic processing. However, recent evidence from developmental studies has begun to blur this sharp distinction. We review evidence for a protracted development of object recognition that is reminiscent of the well-documented slow maturation observed for faces. The prolonged development manifests itself in a retarded processing of metric part relations as opposed to that of individual parts and offers surprising parallels to developmental accounts of face recognition, even though the interpretation of the data is less clear with regard to holistic processing. We conclude that such results might indicate functional commonalities between the mechanisms underlying the recognition of faces and non-face objects, which are modulated by different task requirements in the two stimulus domains.

## Configural Object Recognition

Configural processing can be broadly defined as “any phenomenon that involves perceiving relations between the features of a stimulus” (Maurer et al., 2002, p. 255). In the context of object recognition it is therefore equivalent to the processing of the relations that hold between the parts or components constituting an object. The importance of part relations has been highlighted in Biederman’s influential Recognition-by-components (RBC) model (Biederman, 1987, 2000). According to this model complex objects are encoded as spatial arrangements, or configurations, of basic parts that come from a restricted reservoir of elementary shapes, the so-called geons. Geons are defined by categorical contour properties (like ‘straight’ vs. ‘curved’). Similarly, the spatial configuration of geons is encoded in terms of certain categorical relations (like ‘larger’ vs. ‘smaller,’ or ‘on top of’ vs. ‘besides’). Furthermore, Biederman contrasts coarse shape differences in terms of categorical properties with more subtle ones arising from variations of continuous, or metric, attributes. Again such attributes can be either part-specific (example: a part’s aspect ratio) or part-relational (example: the distance between two parts).

Numerous studies on object processing by children and infants have been inspired by the RBC model. Most have focussed on the status of individual parts. Here parts have been shown to receive particular attention in the analysis of shape similarity (e.g., Tversky and Hemenway, 1984; Saiki and Hummel, 1996) or when categorizing or matching objects (e.g., Madole and Cohen, 1995; Smith et al., 1996). Whether the early primacy of parts in visual processing reflects a peculiar status of geons has, however, remained more contentious (cf. Abecassis et al., 2001; but note Haaf et al., 2003).

Unlike for parts, until recently relatively few studies considered the processing of part relations within the RBC framework. Mash (2006) examined similarity judgements of novel objects consisting of two parts; one of these parts was manipulated in terms of its cross-section (i.e., at part-specific level) and its location relative to the second (i.e., at part-relational level). Young children were found to have a strong bias for classifying objects on the basis of part-specific information only. With increasing age, participants came to use both part-specific and part-relational information in their classification judgements.

Jüttner et al. (2013) asked children aged 7–16 years and adults to judge the correct appearance of familiar animals and artifacts that had been manipulated either in terms of individual parts (for example, by exchanging the head of an animal against that of another animal) or part relations (here: relative size; for example, by changing an animal’s proportions). Both types of manipulation were always calibrated for equal difficulty in adult observers. When detecting part changes even the youngest children performed close to adult levels. By contrast, it was not until 11–12 years that they achieved similar levels of performance with regard to relative size changes, i.e., altered part relations. The developmental dissociation between part-relational and part-specific processing was the same for both types of stimuli thus generalizing similar observations by Davidoff and Roberson (2002). In a further experiment, Jüttner et al. (2013) demonstrated that this dissociation only applied to the recognition of metric changes, not to those at categorical level. They used a set of novel multi-part objects, which permitted precisely controlled manipulations of parts and part relations at categorical or metric level, as defined within the RBC framework. Participants were first trained to associate the novel objects with labels (here: numbers). As in the experiments involving animals and artifacts they then had to judge the correct appearance of these objects when manipulated at part-specific level or that of part relations (here again: the object’s proportions, i.e., the relative size of its parts). For metric manipulations of an object’s proportions, recognition accuracy showed a similarly protracted development as in the case of animals and artifacts. By contrast, no such retardation was observed in the case of categorical changes of an object’s proportions.

**FIGURE 1:**
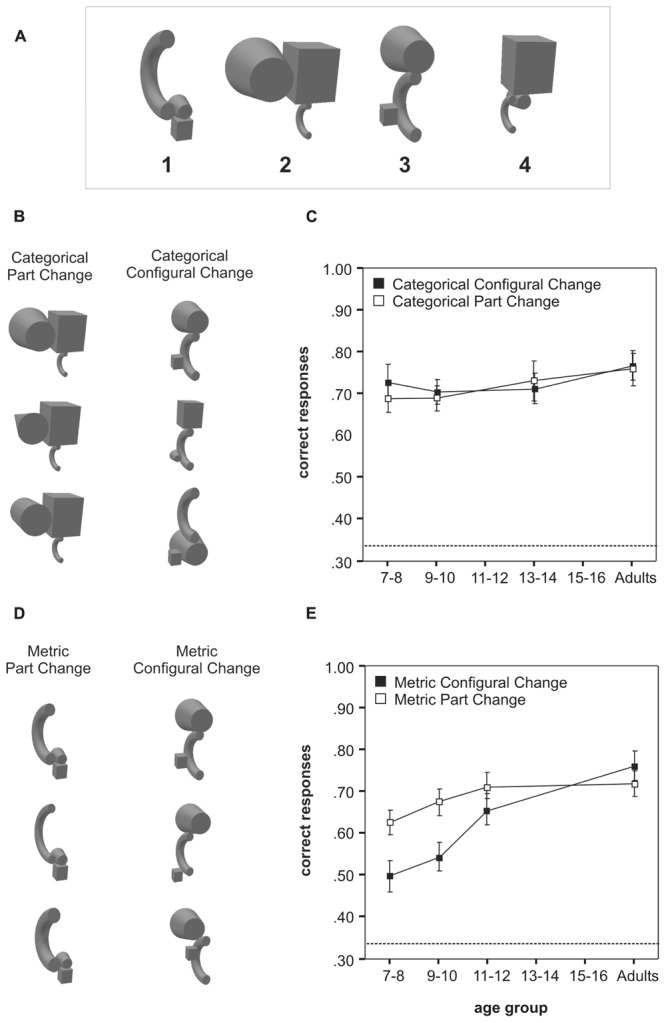
**Development of configural and part-based object recognition for manipulations at categorical and metric level (adapted from Jüttner et al., 2014). (A)** Examples of multi-part objects used in the learning set of Jüttner et al. (2014). Participants were first trained to associate each object with its label (here: the number) during the learning phase of the experiment. **(B)** Examples of multi-part objects used to compare recognition performance for manipulations at categorical level. In each trial of the recognition test, a target object of the learning set (here for illustration purposes always shown in the top row) was presented with two distracters (middle and bottom row). The distracters differed from the target in terms of either a categorical part change (left) or a categorical, configural change of relative part position (right). Participants had to choose the correct depiction of the previously learnt object. **(C)** Mean recognition accuracies as a function of age for the categorical part change and categorical configural change condition. **(D)** As in **(B)** but examples show multi-part objects used to compare recognition performance for manipulations of parts and part relations at metric level. **(E)** Mean recognition accuracies as a function of age for the metric part change and metric configural change condition. Error bars are standard errors. The dashed line at 0.33 indicates chance level.

Using similar stimuli (**Figure 1A**), Jüttner et al. (2014) generalized these findings to the attribute relative position, the second core relational attribute of RBC. Again, even the youngest tested children performed similarly to adults when recognizing categorical changes of individual parts and relative part position (**Figures 1B,C**). By contrast, performance for detecting metric changes of relative part position was distinctly reduced in young children compared to recognizing metric changes of individual parts (**Figures 1D,E**). A similarly late maturation for the processing of metric positional information has been observed in the context of other work involving faces and objects (e.g., Mondloch et al., 2002, 2004; Jüttner et al., 2006; Mash, 2006; Robbins et al., 2011). It has been proposed that the retardation might reflect late developing general perceptual mechanisms (e.g., Crookes and McKone, 2009; Robbins et al., 2011). However, as demonstrated by Davidoff and Roberson (2002) in control experiments involving a paired-comparison task, children’s inability to use part-relations for object recognition cannot be attributed to reduced perceptual discrimination skills. Thus, the reduced sensitivity to metric part-relational information appears to reflect a fundamental limitation concerning the way objects are represented in the memory of the developing mind.

The problems young children have with the detection of subtle positional changes of object parts are reminiscent of the well-documented difficulty they have when assessing spatial relations of facial features. Here it has been shown that children’s sensitivity to detect manipulations of the distances between cardinal features (like eyes, nose and mouth) continues to improve until at least 14 years (Carey et al., 1980; Mondloch et al., 2002; de Heering and Schiltz, 2013). Such processing of spatial relations – also known as second-order processing – can be contrasted with the coarse assessment of the basic spatial layout of facial features – their so-called first-order relations. The sensitivity to the latter develops much earlier and may already be present in newborns (e.g., Goren et al., 1975; Johnson et al., 1991). On this basis it is tempting to draw a parallel between the developmental dissociation for first- and second-order relational processing of facial features on the one hand, and categorical and metric part-relational processing for non-face objects on the other. We will return to this possibility in the final section of our review.

## Holistic Object Recognition

Image-based models of object recognition have been proposed in various forms (e.g., Ullman, 1989; Tarr and Bülthoff, 1995; Riesenhuber and Poggio, 1999). However, they generally assume “holistic” object representations that are “all-in-one” or view-like, and where object features are represented in a quasi-pictorial, two-dimensional coordinate system. While image-based accounts originally were presented as alternative to structural, part-based approaches, later evidence from behavioral (e.g., Hummel, 2001; Hayward, 2003; Thoma et al., 2004) and neuroimaging (e.g., Vuilleumier et al., 2002; Thoma and Henson, 2011) studies suggests that structural and image-based representations might co-exist in the visual system. This idea has been most comprehensively formulated in Hummel’s (2001) dual-route model. It proposes that objects are processed in two different formats – analytic and holistic – that are combined into a hybrid representation in long-term memory. The analytic pathway involves explicit structural descriptions, employing a dynamic, attention-driven binding mechanism that operates on an object’s parts and their relations – similar to Biederman’s RBC model. By contrast, the holistic pathway is view-like and involves a static, attention-independent binding of an object’s local shape features via their relative location in a so-called surface-map. The surface map preserves topological relations of these features, resulting in a template-like, holistic representation.

So far, research assessing the relative extent to which the holistic and analytic route contribute to object recognition in children has been scarce. In the one known study, Wakui et al. (2013) tested holistic and analytic recognition performance for everyday objects in 7- to 12-year-old children and adults. They used a repetition priming paradigm that involved two briefly presented prime stimuli: one attended and the other ignored. Priming was assessed in terms of the facilitation for naming a subsequently presented probe stimulus. According to the dual-route model, holistic priming should in principle be observed both for the attended and the ignored prime stimulus. However, given the view-like object representation used by the holistic route the priming should critically depend on the pictorial identity of prime and probe. By contrast, analytic priming should result only from the attended prime stimulus. Due to the more abstract object format implied by the analytic route, such priming should tolerate image differences between probe and prime as long as those permit at least a partial matching of the underlying structural representations. In Wakui et al.’s (2013) study, adults showed both holistic and analytic priming, in accordance with previous work (e.g., Stankiewicz et al., 1998; Thoma et al., 2004, 2007). By contrast, the data for children only demonstrated analytic but no holistic priming, suggesting a developmental primacy for part-based over holistic object recognition.

A few other studies have assessed children’s ability for holistic object perception by employing paradigms more typically used to test holistic processing of faces. Cassia et al. (2009) compared composite effects for faces and non-face objects in 3- to 5-year-old children and adults. The study involved a matching task between composites constructed from the top and bottom halves of faces and non-face stimuli (here: frontal images of cars). A composite effect, suggestive of holistic processing and indicated by an impaired matching performance when the stimulus halves were spatially aligned relative to a condition when they were not aligned, was found for faces in children as young as 3 years. By contrast, no evidence of holistic processing was observed for non-face objects in any of the tested age groups. Meinhardt-Injac et al. (2014) used a context congruency paradigm to compare the processing of faces and non-faces (here: watch faces) in children aged 8–16 years and adults. For both types of stimuli, observers had to make a same/different judgment regarding the internal features of two test stimuli while their (unattended) external features differed in terms of congruency – they could either agree or disagree. With increasing age task performance improved more slowly for faces than non-face objects. However, holistic processing, as assessed by the impact of context congruency, was only observed for faces but not for watches.

The interpretation of the findings of Cassia et al. (2009) and Meinhardt-Injac et al. (2014) is complicated by the fact that for non-face stimuli no holistic processing was observed in adult observers. A possible explanation could be the requirement of structural long-term representations for holistic effects to become manifest (Davidoff and Donnelly, 1990; Donnelly and Davidoff, 1999). In the absence of such representations, as might be the case in non-experts for clock faces (Meinhardt-Injac et al., 2014) and fronts of cars (Cassia et al., 2009), adults – and children – may have predominantly relied on part-based information to perform the tasks.

Despite such methodological challenges, the current evidence suggests that holistic processing develops distinctly earlier for faces than objects. For the former, such processing has been reported for children as young as 4 (e.g., Carey and Diamond, 1994; Tanaka et al., 1998; Pellicano and Rhodes, 2003; de Heering et al., 2007; Cassia et al., 2009) even though its maturational progression remains controversial (e.g., Crookes and McKone, 2009; but note Schwarzer et al., 2010). For non-face objects, holistic processing so far has only been reported in adults; for children, this kind of processing appears not to emerge before late adolescence.

## Toward a Common Framework for the Processing of Faces and Non-Face Objects

**FIGURE 2:**
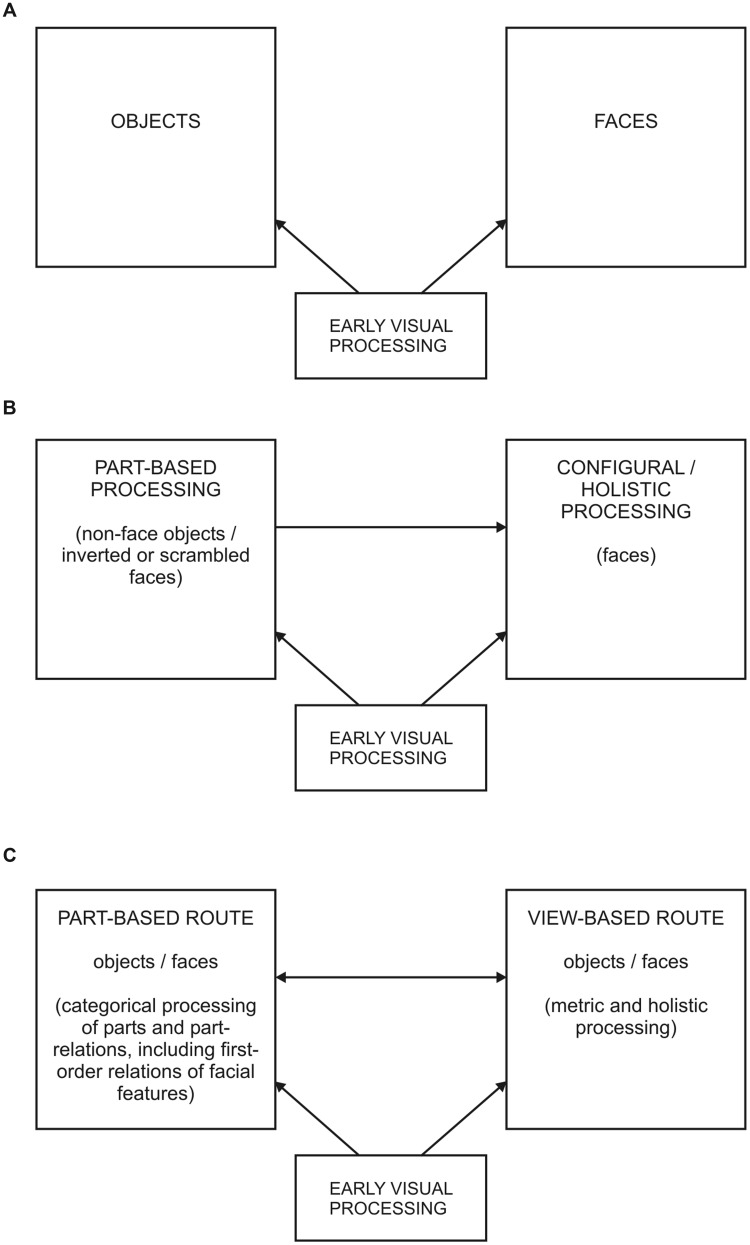
**Three different models relating object and face perception. (A)** According to the model by Farah (adapted from Farah, 1996) object and face processing are largely independent from each other. **(B)** Holistic/part-based model of Piepers and Robbins (adapted from Piepers and Robbins, 2012). Here face perception is assumed to be supported by both part-based and configural/holistic processing whereas object perception is only part-based. **(C)** Proposed dual-route framework for the processing of objects and faces, consisting of a categorical, part-based and a metric, view-based pathway. The two routes operate in parallel but can also augment each other. Their relative contribution is assumed to depend on stimulus domain as well as on task, and to be modulated by developmental progression.

In this review we have discussed recent findings regarding configural and holistic object processing that suggest a more intricate relationship between the perception of objects and faces than previously postulated. As outlined in the introduction, over the last two decades the notion of a quasi-dichotomy of object and face perception, illustrated in **Figure 2A** by Farah’s (1996) early model, has given way to more differentiated accounts. These acknowledge the potential contribution of parts to recognition in both stimulus domains, as demonstrated by the model of Piepers and Robbins (2012) in **Figure 2B**. Based on the evidence presented in the preceding sections we propose that this relationship may be even closer. Combining elements of Hummel’s (2001) dual route model with those of the holistic/part-based account of Piepers and Robbins, **Figure 2C** shows the first sketch of a new, common framework for the processing of faces and objects.

The proposed framework comprises two parallel pathways: (1) a part-based route which in the case of objects encompasses a structural (analytical) description of parts and part relations at categorical level, in the case of faces a representation of the first-order relations of facial features; (2) a view-based route which both for objects and faces includes a metric, template-like representation supporting holistic processing. It is further assumed that part-based and view-based route interact and support each other. For example, part-based information may affect view-based processing as illustrated by the impact of feature shape on holistic face perception (McKone and Yovel, 2009). Conversely, view-based representations may augment part-based descriptions, facilitating the metric processing of parts and their relations in the case of objects, and second-order configural processing in the case of faces. At the level of holistic processing of objects and faces, such facilitation may underlie the part-whole effect, i.e., the superior identification performance for a part shown in the context of a complete stimulus than when shown in isolation (Davidoff and Donnelly, 1990; Tanaka and Farah, 1993; Donnelly and Davidoff, 1999).

Both object and face recognition are assumed to show a developmental transition from a coarse, categorical representation based on parts and their relations to a dual format that is augmented by a metric, view-based representation. However, the developmental trajectory of this transition differs between the two stimulus domains – possibly driven by different task demands: subordinate identification in the case of faces, basic-level recognition in the case of objects. For faces, categorical representations accounting for the very early, if not innate, sensitivity to first-order relations of facial features may soon be augmented by a view-based representation facilitating an onset of holistic face perception in early infancy (e.g., Tanaka et al., 1998; Cassia et al., 2009). By contrast, for non-face objects a categorical representation based on parts and their relations may remain the preferred format until late adolescence. This is suggested by part-primacy effects found in children for categorization and similarity judgements (e.g., Madole and Cohen, 1995; Smith et al., 1996) as well as the early maturation of categorical part-relational processing (Jüttner et al., 2013, 2014). For both stimulus classes, the spatial precision of view-based representations may improve throughout adolescence. The prolonged maturation for metric configural and holistic processing observed for faces (e.g., Schwarzer et al., 2010; Kadosh, 2012; Meinhardt-Injac et al., 2014) and objects (Jüttner et al., 2013, 2014; Wakui et al., 2013) supports this view.

The dual-route framework shown in **Figure 2C** does not necessarily argue for a neuro-functional isomorphism of face and object recognition. A category specificity for faces and objects in the adult brain could in principle imply separate dual representations within the well-established functional core regions of the respective stimulus domain, like the fusiform face area (FFA) and the occipital face area (OFA) in the case of faces, and the lateral occipital complex (LOC) in the case of objects. However, recent evidence from developmental neuroimaging studies also raises the possibility that the processing routes for faces and objects may overlap. In particular the developmental trajectory of face specificity within the fusiform gyrus continues to be controversial. While a few studies have reported a mature activation of the FFA in children as young as 4 years (Pelphrey et al., 2009; Cantlon et al., 2011) the majority observed significant developmental changes through mid and late adolescence (e.g., Gathers et al., 2004; Golarai et al., 2007; Scherf et al., 2007, 2011; Haist et al., 2013). Thus, the face specificity of the FFA may emerge gradually as a consequence of the particular task demands of face identification (cf. also Scherf et al., 2011), leaving room for a potentially shared processing of faces and objects in categorization tasks at basic level.

Conceptually, such a partially shared processing of faces and objects could be placed at the structural encoding stage of Bruce and Young’s (1986) classical model of face perception. According to Bruce and Young’s original account this stage encompasses part-specific and part-relational processing as well as the (basic-level) classification of a stimulus as a face. Based on the evidence presented in this review we propose that it might be better described in terms of our dual-route framework, and underlie the basic-level categorization of both faces and objects. Information from that stage might then feed into separate, domain-specific modules that accommodate the different requirements of face and object recognition at subordinate level. Future work will need to further clarify the relative contribution of the two routes in our framework across tasks and stimulus domains, as well as their neurological basis.

## Author Contributions

All authors listed, have made substantial, direct and intellectual contribution to the work, and approved it for publication.

## Conflict of Interest Statement

The authors declare that the research was conducted in the absence of any commercial or financial relationships that could be construed as a potential conflict of interest.
